# Using Dimensionality Reduction to Analyze Protein Trajectories

**DOI:** 10.3389/fmolb.2019.00046

**Published:** 2019-06-19

**Authors:** Gareth A. Tribello, Piero Gasparotto

**Affiliations:** ^1^Atomistic Simulation Centre, School of Mathematics and Physics, Queen's University Belfast, Belfast, United Kingdom; ^2^Department of Physics and Astronomy, Thomas Young Centre, University College London, London, United Kingdom

**Keywords:** molecular dynamics, dimensionality reduction, machine learning, trajectory analysis, computer simulation, clustering

## Abstract

In recent years the analysis of molecular dynamics trajectories using dimensionality reduction algorithms has become commonplace. These algorithms seek to find a low-dimensional representation of a trajectory that is, according to a well-defined criterion, optimal. A number of different strategies for generating projections of trajectories have been proposed but little has been done to systematically compare how these various approaches fare when it comes to analysing trajectories for biomolecules in explicit solvent. In the following paper, we have thus analyzed a molecular dynamics trajectory of the C-terminal fragment of the immunoglobulin binding domain B1 of protein G of Streptococcus modeled in explicit solvent using a range of different dimensionality reduction algorithms. We have then tried to systematically compare the projections generated using each of these algorithms by using a clustering algorithm to find the positions and extents of the basins in the high-dimensional energy landscape. We find that no algorithm outshines all the other in terms of the quality of the projection it generates. Instead, all the algorithms do a reasonable job when it comes to building a projection that separates some of the configurations that lie in different basins. Having said that, however, all the algorithms struggle to project the basins because they all have a large intrinsic dimensionality.

## 1. Introduction

For many years researchers have sought to determine whether it is possible to predict the tertiary structure of a protein from the amino acid sequence alone. Numerous structure prediction algorithms have been developed to solve this problem and these algorithms have then been tested in the biennial, community-wide blind tests to predict the unknown structures of proteins. The tertiary structure of the protein is only a part of the story, however. To truly understand how these molecules function in the cell we must also understand their dynamical behavior (Dunker et al., 2008; Constanzi, 2010; Goldfeld et al., 2011; Kmiecik et al., 2015). In fact, a whole new class of intrinsically disordered proteins (IDP) that do not have the same familiar and relatively permanent tertiary structures has been discovered (Dyson and Wright, 2005).

Molecular dynamics (MD) simulations with force fields that model the interactions between the atoms in the biomolecule have emerged as a useful tool for investigating the dynamical structure of proteins. This technique is, in fact, particularly important for IDPs as the experiments alone often do not provide sufficient information on the conformers adopted by the biomolecules. Detailed structural information is thus obtained by formulating constraints based on the experimental data and by then performing constrained MD simulations (Bonomi et al., 2017). There is a problem, however, when it comes to visualizing the results from these MD simulations. Biomolecules, unlike simpler chemical systems such as solids, do not undergo transitions that involve a change of symmetry. Instead, they undergo transitions between various low symmetry structures, which makes it difficult to know how to analyze the trajectories that emerge from MD simulations.

During the last few decades, many researchers have sought to solve the problems outlined in the previous paragraph by analyzing their MD trajectories using dimensionality reduction algorithms. The theory behind such approaches is that the computer can determine what features of the data are important and what features are simply noise. Many different algorithms have been used to analyze MD trajectories and some have even been developed with this particular purpose in mind (Garcia, 1992; Amadei et al., 1993; Balsera et al., 1996; Yuguang et al., 2005; Das et al., 2006; Konrad, 2006; Plaku et al., 2007; Spiwok et al., 2007; Zhuravlev et al., 2009; Stamati et al., 2010; Sutto et al., 2010; Ceriotti et al., 2011; Spiwok and Kralova, 2011; Tribello et al., 2012; Noé and Clementi, 2015, 2017; Tiwary and Berne, 2016; Sultan and Pande, 2017; Chen and Ferguson, 2018; Sultan et al., 2018). Much less work has been done, however, to compare the performance of the various dimensionality reduction algorithms although there are a few notable examples of work on systems in implicit solvent in the literature (Duan et al., 2013).

One reason why few systematic comparisons between the projections of trajectories generated using different dimensionality reduction have been performed is that it is difficult to formulate an appropriate method to test the quality of a projection. After all, if we knew what the appropriate method for analyzing our trajectory was we most likely wouldn't be reliant on dimensionality reduction algorithms. Duan et al. (2013) argue that one feature of a good projection is that the distances between the projections of the points are similar to the true distances between the trajectory frames. This criterion is undoubtedly sensible but it is also the criterion that is used when optimizing the projection. What we thus find out when it is measured is the extent to which the algorithm was able to satisfy the constraints of the optimization problem. As Duan et al. (2013) point out it is much more difficult to unequivocally say that the assumptions of method X are appropriate. Particularly so when it comes to the non-linear methods. Nevertheless, in what follows we use a number of different algorithms to analyze the trajectory of a biomolecule in explicit solvent. We show two-dimensional projections of the trajectory that are obtained using each of these algorithms and perform various analyses to compare how well these projections have encoded the information in the trajectory in section 3. Before getting onto this, however, we provide some background information on the various algorithms that we have used in section 2 and the trajectory we have analyzed in section 3.

## 2. Background

A molecular dynamics trajectory for a set of *N* atoms is essentially an ordered set of 3*N*-dimensional vectors. Furthermore, if we assume that the simulated system is equilibrated and if we are only interested in static properties then the order the vectors are in is not particularly important. The problem of analysing the trajectory thus reduces down to one of simply visualizing the position of each frame in the trajectory relative to all the others. Obviously, however, we cannot draw a diagram illustrating the position of each trajectory frame in the 3*N*-dimensional vector space of atomic positions and are thus forced to discard (ideally) all but two of these dimensions.

Oftentimes decisions as to how to plot the relationships between the trajectory frames are made using chemical or physical intuition about the problem under study. In these cases, some function/s of the atomic positions - usually referred to as collective variables or CVs - is computed for each of the trajectory frames. The positions of each of the trajectory frames in the low-dimensional CV space can then be plotted so that conclusions can be drawn about the parts of space that were sampled in the trajectory.

Dimensionality reduction algorithms adopt a similar approach. Instead of using chemical/physical intuition to decide on the appropriate low dimensional space in which to visualize the data, however, dimensionality reduction algorithms introduce a loss function. Optimization algorithms are then used to ensure that a low-dimensional representation of the data that minimizes the value of this loss function is found.

To understand how these algorithms work in practice consider how the multidimensional scaling (MDS) algorithm (Borg and Groenen, 2005) would produce a low dimensional representation, {**x**^(*i*)^} of the *N* *M*-dimensional vectors in the set, {**X**^(*i*)^}. The first step is to compute the dissimilarity between each pair of trajectory frames using Pythagoras theorem:

(1) $$||X^{\left(i\right)}-X^{\left(j\right)}||=\sqrt{\sum_{k=1}^{M}{\left(X_{k}^{\left(i\right)}-X_{k}^{\left(j\right)}\right)}^{2}}$$

The low dimensional representation is then found by optimizing the loss function:

(2) $$\chi (\{x^{\left(i\right)}\})=\sum_{i\neq j}{\left\{\left||X^{\left(i\right)}-X^{\left(j\right)}||-||x^{\left(i\right)}-x^{\left(j\right)}|\right|\right\}}^{2}$$

where **x**^(*i*)^ and **x**^(*j*)^ are the low dimensional representations of points *i* and *j* respectively. In other words, the MDS algorithm works by endeavoring to arrange the points in the low dimensional space so that the distances between the projections are the same as the dissimilarities between the trajectory frames.

All of the dimensionality reduction algorithms that have been used to analyze molecular dynamics trajectories work using a strategy that is similar to the one described above. In short, some features that describe how the data is arranged across the high dimensional space are computed. Points are then arranged in the low dimensional space in a way that reproduces the high-dimensional features as closely as possible. A large number of algorithms exist, however, because one can perform numerous variations on this theme. The best way to understand these variants is to consider the choices that must be made in order to analyze a trajectory using one of these algorithms:

- **How to represent the trajectory frames** - The simplest representation to use for the trajectory frames is a list of atomic coordinates. Using the atomic coordinates is not particularly sensible, however, as one essentially discards all chemical and physical intuition about the problem at hand. It is thus often better to embed the known physical details about the problem when one is calculating the high-dimensional vectors that represent each of the trajectory frames. As a case in point, it may well be better to input vectors of backbone dihedral angles into the dimensionality reduction algorithm when one is examining a trajectory of a biomolecule. Alternatively, a number of general purpose representations of atomic structures have been developed in the context of fitting potentials based on the results of density functional theory calculations (Behler, 2011; Bartók et al., 2013; Willatt et al., 2018). These representations have the advantage of providing a systematically convergent description of the chemical environment and were used in De et al. (2016); Bartók et al. (2017); Musil et al. (2018).
- **Whether to use landmark points and if so how to choose these landmarks** - Dimensionality reduction algorithms scale quadratically with the number of input vectors. It is thus often not feasible to perform a dimensionality reduction with a whole trajectory as input. Researchers therefore often adopt a more computationally-efficient strategy whereby they run the algorithm on a small number of so-called landmark frames. Projections for all the frames in the trajectory are then constructed using an out-of-sample procedure. Obviously, using this procedure entails choosing an appropriate number of landmark points and devising a strategy for selecting these landmarks. Typically, however, one of two strategies is employed either (i) landmarks frames are selected randomly so the distribution of landmarks resembles the distribution of trajectory frames or (ii) landmarks frames are selected using farthest point sampling so as to have frames from all the regions of configuration space that were explored. A third option that combines the strengths of these two approaches is discussed in Ceriotti et al. (2013)
- **How to construct the loss function** - By changing the way the loss function is defined one can change what features from the high-dimensional space the algorithm is endeavoring to reproduce as it arranges the projections in the low-dimensional space. Minimizing Equation 2 for instance is akin to attempting to reproduce the Euclidean distances between the high-dimensional vectors. It is possible to use a different method for calculating the dissimilarity between the trajectory frames, however. For example, in isomap (Tenenbaum et al., 2000) dissimilarities between the high-dimensional frames are computed by using Dijkstra's shortest path algorithm to compute the approximate geodesic distances between frames. In an isomap projection, it is thus the geodesic distances between frames that are reproduced. Other algorithms, sketch-map for example (Ceriotti et al., 2011), have a loss function that is designed so that only a particular subset of the dissimilarities between the trajectory frames are reproduced. Yet another option is to design the loss function so that a matrix of non-linear kernels is reproduced rather than a matrix of dissimilarities (Schölkopf et al., 1998; Schölkopf et al., 1999). One final option is to design a loss function that reproduces the distribution of points in the neighborhood of each of the high-dimensional points (van der Maaten and Hinton, 2008).
- **How to optimize the loss function** - As with any optimization problem there is a concern when finding the low dimensional projection that the minimum found is a local rather than the global optimum. In many of the commonly used algorithms, this problem is sidestepped by insisting that the distances between the projections should constitute the best linear approximation of the dissimilarities. This approximation simplifies matters considerably as finding the projections simply becomes a matter of diagonalizing a matrix. The fact remains, however, that the algorithm used to optimize the loss function may have an effect on the final projection produced.

The rationale that should be born in mind when making these decisions is not always clear. In other fields, decisions are often made based on an understanding of what the high-dimensional data looks like and on an understanding of what features in the high-dimensional data set the users of these algorithms would like to reproduce (Rosman et al., 2010). One might therefore, suspect that by trying a range of algorithms and by determining how well each one performs one might be able to get some insight into the structure of the data in the high dimensional space.

## 3. Methods

To test how effective various dimensionality reduction algorithms are at projecting data from biomolecular trajectories we took the data from the parallel tempering metadynamics trajectories of the 16-residue C-terminal fragment of the immunoglobulin binding domain B1 of protein G of Streptococcus that was generated in Ardevol et al. (2015) and projected it using a range of different algorithms. Within that work, the protein was simulated using Gromacs-4.5.5 (Hess et al., 2008), the AMBER99SB-ILDN* force field (Lindorff-Larsen et al., 2010) and an explicit solvation model. The protein and surrounding water molecules were then simulated for 300 ns/replica with metadynamics biases that acted on the radius of gyration and the number of hydrogen bonds between backbone atoms. The same protein was studied in the work in the work on comparing different dimensionality reduction algorithms by Duan et al. (2013) but an implicit solvent model was used in that work rather than the explicit model that we have used.

The wild-type trajectory in the work of Ardevol et al. (2015) that we have analyzed in this work contains 150,000 trajectory frames. Running each of the dimensionality reduction algorithms on this large number of trajectory frames would be computationally prohibitive so we selected a subset of 25,311 to analyse with each of the algorithms by sampling configurations from the trajectory of the lowest-temperature replica at random. For each of these configurations, we computed the full set of 32 torsional backbone dihedral angles. Two-dimensional projections for each of these 32-dimensional vectors were then generated using the implementations of the various algorithms that are available in SciKit Learn (Pedregosa et al., 2011) and the sketch-map code (Ceriotti et al., 2011). Detailed step-by-step instructions showing how these projections were generated using these tools can be found in the supporting information (Data Sheet 1).

The algorithms that we used to project the data were principal component analysis (PCA) (Jolliffe, 2002), Laplacian Eigenmaps (Belkin and Niyogi, 2003), isomap (Tenenbaum et al., 2000), t-distributed stochastic neighbor embedding (t-SNE) (van der Maaten and Hinton, 2008) and sketch-map (Ceriotti et al., 2011). In addition, we also generated a projection by simply minimizing Equation 2 using conjugate gradients (dist. match).

We performed PCA on the dihedral angles in a way that is sympathetic to the periodicity of these variables by using the method described in Yuguang et al. (2005); Konrad (2006); Altis et al. (2007). Meanwhile, for all the remaining algorithms we simply incorporated periodic boundaries when calculating the vectors connecting the positions of the trajectory frames in the space of dihedral angles. For isomap and Laplacian Eigenmaps we constructed graphs in the high dimensional space by connecting each point to its 15 nearest neighbors. For Laplacian Eigenmaps we then used a Gaussian kernel with a gamma parameter of 1. When using t-SNE we employed the Barnes-Hut implementation with a perplexity of 110 and a theta value of 0.5. Lastly, for sketch-map, we selected 1000 landmark point using the well-tempered farthest point sampling algorithm that is described in the appendix of Ceriotti et al. (2013) and a gamma parameter of 0.1. Weights for each of these landmarks were generated using a Voronoi procedure and the sketch-map stress function with parameters σ = 6, *A* = 8, *B* = 8, *a* = 2 and *b* = 8 was then optimized to find projections. Once projections for these landmarks had been found the rest of the trajectory was projected using the out of sample procedure described in Tribello et al. (2012).

## 4. Results

**Figure 1:**
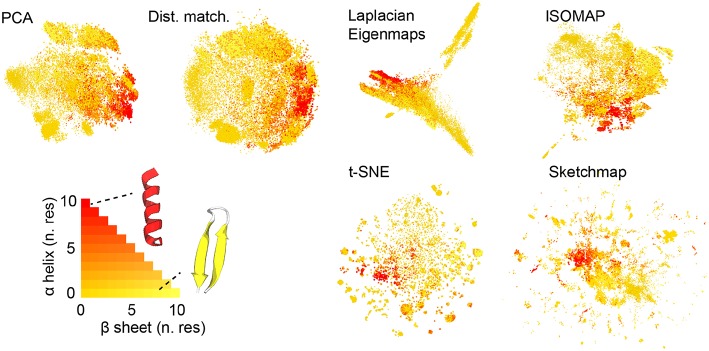
The projections of the trajectory that were generated using each of the various dimensionality reduction algorithms. As discussed in the text the projections in these figures are colored in accordance with the secondary structure content of the corresponding trajectory frame. As you can see the majority of the algorithms do a reasonable job of separating those frames that have a configuration that resembles an alpha helix from those that have a configuration that resembles a beta sheet.

Figure 1 shows the projections of the trajectories for immunoglobulin binding domain B1 of protein G that we obtained. Before projecting the trajectory we used the STRIDE algorithm discussed in Frishman and Argos (1995) to determine the secondary structure content in each of the frames that were analyzed. In particular, we counted the number of residues that had a structure that was similar to an alpha helix and the number of residues that had a structure that was similar to a beta sheet. When constructing the projections in Figure 1 we thus colored the projections according to the number of residues in the corresponding trajectory frames that appeared to be in an alpha helix configuration and the number of residues that appeared to be in a configuration that resembled a beta sheet. Coloring the projections in this way gives us a qualitative way to compare how well each of the algorithms does when it comes to projecting the trajectory data. What we see is that all the algorithms do a reasonable job of separating the configurations that are predominantly alpha-helix-like from those that have a structure that is predominantly composed of beta sheets. In this sense at least then the algorithms all give a reasonable projection of the high-dimensional data.

**Figure 2:**
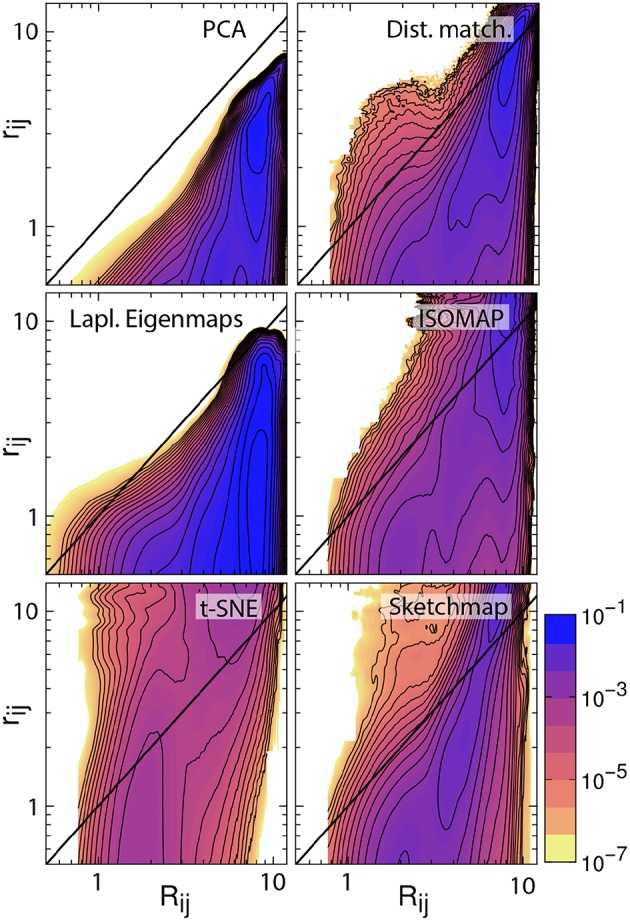
Histograms illustrating the joint probability density function for the dissimilarities between the configurations in the trajectory and the distances between the corresponding projections of these trajectory frames. The particular projections that have been analyzed here are those that are shown in Figure 1. The black line in each of these figures is the line *R*_*ij*_ = *r*_*ij*_. For an ideal projection all the density in these histograms would lie on this line.

There are additional observations to be made based on the results in Figure 1. For PCA the distances between the projections are systematically shorter than the dissimilarities between the corresponding trajectory frames. This fact is illustrated in Figure 2, which shows pair distribution functions for the dissimilarities, *R*_*ij*_, between the frames and the distances, *r*_*ij*_, between their corresponding projections for each of the representations of the data shown in Figure 1. To compute the dissimilarities, *R*_*ij*_, we inserted the vector of backbone dihedral angles for each pair of trajectory frames into Equation 1 and made suitable dispensations for the periodicity of these variables. The distances, *r*_*ij*_, were, meanwhile, computed by using Equation 1 on the projections of these points. With these two quantities computed we then determined these probability density functions by using:

$$P(D,d)=\frac{1}{0.5N(N-1)}\sum_{i=2}^{N}\sum_{j=1}^{i}\delta (R_{ij}-D)\delta (r_{ij}-d)$$

where *N* is the number of frames that were projected and where δ is a Dirac delta function.

If a projection is perfect the distances between the projections and the dissimilarities between the trajectory frames are identical and all the density in the joint distribution function is concentrated on the line *y* = *x*. For all the probability density functions shown in Figure 2, however, we see that the density away from *y* = *x* is substantial and we, therefore, know that the projections are thus imperfect. Furthermore, for PCA we see that all the density lies underneath the line *y* = *x*, which is why we are able to state that the distances between the projections are systematically shorter than the dissimilarities between the corresponding trajectory frames. The fact that these distances are shorter when we use this algorithm is unsurprising, however. This algorithm projects the high-dimensional data into a linear subspace. Any differences between configurations that are along directions that are orthogonal to this subspace are thus discarded when projections are constructed in this way.

It is possible to formulate PCA as a linear optimization of Equation 2. In other words, this algorithm projects the data in the linear subspace that is best able to reproduce the dissimilarities between frames, which, incidentally, is why the distances between the projections are systematically shorter than the dissimilarities between the trajectory frames. We can avoid producing a projection in which the distances between points are systematically shorter than the dissimilarities between the corresponding trajectory frames by minimizing Equation 2 using an iterative algorithm such as conjugate gradient. The top right panel of Figure 2 illustrates that when we do so the joint probability distribution for the distances and dissimilarities is then peaked around the line *y* = *x* so some distances are projected further apart than they should be while others are projected closer together. The effect this has on the appearance of the projection is illustrated in Figure 1. Essentially the projections of the points are spread more uniformly over the low dimensional space than they would be if the projection had been constructed using PCA. Given that one of our aims is to identify the various basins in the free energy landscapes that were sampled during the trajectory this spreading out of the projections is clearly disadvantageous as it may cause different basins to overlap with each other.

The other algorithms that have been tested in Figures 1, 2 all use different criteria when constructing the projections. In other words, these algorithms do not seek to generate projections in which the Euclidean distances between the trajectory frames are reproduced in the projection. Instead, Laplacian Eigenmaps (Belkin and Niyogi, 2003) and isomap (Tenenbaum et al., 2000) seek to reproduce the diffusion distances and geodesic distances respectively and calculate these distances by using ideas from graph theory. t-SNE (van der Maaten and Hinton, 2008) and sketch-map (Ceriotti et al., 2011), meanwhile, do not try to generate projections in which the distances are reproduced at all. Instead, sketch-map seeks to ensure that points that are far apart in the high-dimensional space are projected far apart, while simultaneously ensuring points that are close together in the high-dimensional space are projected near to each other. t-SNE, meanwhile, endeavors to generate a low-dimensional projection that reproduces the distribution of neighbors around each point in the high-dimensional space. The projections generated using each of these algorithms that are shown in Figure 1 differ starkly from those that are generated using the two forms of distance matching that were described previously. Reassuringly, however, all four algorithms do a reasonable job when it comes to separating the configurations that resemble an alpha helix from those that resemble a beta sheet.

The non-Euclidean-distance-matching algorithms: isomap, Laplacian Eigenmaps, t-SNE, and sketch-map make assumptions about the structure of the manifold from which the trajectory frames are sampled, which may or may not be valid. These assumptions affect the dissimilarities that these algorithms attempt to reproduce and can thus affect the distances between the projections. In fact, Duan et al. (2013) showed that projections generated using isomap do not preserve the neighborhood structure in the high dimensional space even when 50-dimensional projections are constructed precisely because of the way in which the geodesic distances are constructed. Furthermore, Brown et al. (2008) showed that these algorithms can give incorrect estimates for the dimensionality of energy landscapes precisely because they assume that a manifold-like structure exists that may not be there. To investigate the effect these assumptions are having on the appearance of the projections the bottom four joint probability distributions in Figure 2 illustrate how each of the non-distance-matching algorithms performs when it comes to generating a projection in which the Euclidean distances between the various trajectory frames are reproduced. As you can see only Laplacian Eigenmaps produces a projection in which the distances between projections are systematically shorter than the dissimilarities between the corresponding trajectory frames. All the remaining algorithms generate projections in which only some distances are underestimated. The distances between the projections of the points in these representations are, in contrast to the other algorithms, predominantly larger than the dissimilarities between the corresponding trajectory frames. This “stretching out" of the distances in the projections is arguably a good thing as it ensures that the representations of the various basins in the low dimensional space do not overlap. At the same time, however, it may be that this stretching out of space causes basins to appear split into smaller pieces in the projection, which may give one the impression that there are more features in the energy landscape than there are in actuality.

**Figure 3:**
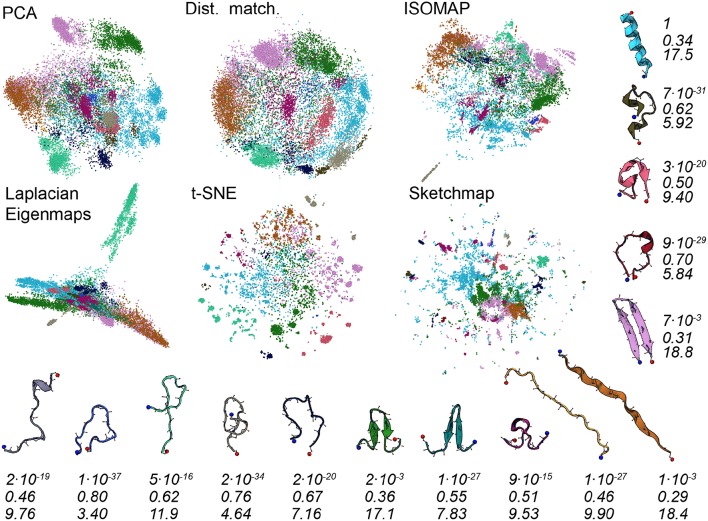
As discussed in the text, we used the PAMM clustering algorithm to identify clusters that correspond to free energy minima in the high dimensional space. In this figure, we have thus shown the projections again but this time with the points colored according to the particular cluster the PAMM algorithm identifies each of the high dimensional configurations to lie within. Representative and similarly-colored configurations from each of the various clusters are shown below and to the right of the projections. The three rows of numbers beneath the representative configurations provide information on the covariance matrix for each of the clusters that PAMM identified. The top row of numbers contains the determinants of this matrix but these numbers have been scaled so that the cluster with the largest determinant has a determinant of 1. To compute the second row of numbers, meanwhile, we divided the sum of the first two eigenvalues of these covariance matrices by the sum of all the eigenvalues of these matrices. The final row of numbers contains the estimated dimensionality of each of the clusters, which was computed using Equation 3.

To better understand the various projections in Figure 1 we performed an analysis of the trajectory that was similar to that performed in Gasparotto et al. (2018). To generate the images shown in Figure 3 we analyzed the high-dimensional data using the probabilistic analysis of molecular motifs (PAMM) method that is discussed in Gasparotto and Ceriotti (2014) and Gasparotto et al. (2018). This clustering method works by first selecting a sparse grid of points in the high dimensional space. The probability density at each of these grid points is then computed using kernel density estimation (KDE). Once the density at each of these points has been estimated the Quick-Shift algorithm is used to connect points on the grid to nearby points that have higher probability densities unless a stopping criterion is satisfied. The points at which the stopping criteria are satisfied are then assumed to correspond to the various local maxima in the probability density. In Gasparotto et al. (2018) dimensionality reduction was performed in order to better understand the clusters output by PAMM. In this work, however, we performed PAMM in order to assign each of the structures in our trajectory to the nearest local maximum in the high-dimensional probability distribution so that the way in which each of these features is represented in each of the projections could be visualized. Figure 3, therefore, shows representative configurations from each of the eleven modes that were identified using PAMM. The projections in Figure 3 are then colored according to the particular mode from which the corresponding trajectory frame was sampled. Once again we find that all the algorithms do a reasonable job of projecting the data. In most of the projections, the different modes are projected in different parts of the low dimensional space and there is little overlap between the projections of the modes.

There are a number of specific things that are worth noting about the projections shown in Figure 3. The first is that all the algorithms clearly struggle to project the PAMM motif that is shown in light blue in the figures. In all the projections the blue points are split into multiple distinct clusters. If, as PAMM is telling us, these points are all from the same mode you would hope that they would all be clustered in together in a single feature of the projection. In other words, you would hope that the points that are clustered together in the high-dimensional space would appear clustered together in the projection. Given that this appears to not be the case it would be unwise to run a clustering algorithm on the low-dimensional projection.

The fact that the blue PAMM motif appears to be so diffuse in the projections is surprising as if you look at the representative structure at the top right of the figure the structures in this basin would appear to resemble alpha helices. One would expect such a structure to be in a deep energetic minimum in the free energy landscape and one might further expect that the entropy of the configuration - and hence the size of the feature - to be small. A comparison of Figures 1 and 3 clears these matters up, however. In Figure 1, remember, the points were colored red if they had a configuration resembling an alpha helix. It is clear that the red regions in Figure 1 are considerably smaller than the blue regions in Figure 3. It is thus clear that the mode that is colored light blue in Figure 3 must also contain structures that do not have a configuration that resembles an alpha helix. What thus seems likely is that the alpha helix configuration is at the bottom of a broad funnel in the free energy landscape, which is why these features appear to be so diffuse in these projections.

The fact that the alpha helix lies at the base of a broad funnel in the free energy landscape is further confirmed by the numbers that are given underneath the representative configurations in Figure 3. These numbers were computed from the covariance matrices for each of the various clusters that were identified using PAMM. The first row of numbers in Figure 3 gives the determinant of the covariance matrices scaled by the determinant of the covariance of the largest cluster. As you can see the alpha-helical cluster that is shown in light-blue has the largest determinant and this cluster is thus the mode that takes up the largest volume of the high-dimensional space by some considerable margin. Furthermore, the second largest mode is the other folded configuration; namely, the purple-colored basin that includes the beta-hairpin configuration.

To compute the second row of numbers in Figure 3 we diagonalized the covariance matrix for each of the PAMM clusters and calculated the sum of the largest two eigenvalues of this matrix divided by the sum of all the eigenvalues. These numbers thus give a measure of how much of the variance of each particular feature can be represented in a two-dimensional linear subspace of the high dimensional space. It is apparent from this analysis that each of the PAMM features that we have identified is not well represented in a two-dimensional space as much of the variation within each of the basins is in directions that are orthogonal to these two principal eigenvectors. We should thus perhaps not be surprised to find that the algorithms struggle to project these clearly-high-dimensional features correctly.

Further information on the features that are difficult to reproduce in a two-dimensional space is given in the third row of numbers in Figure 3. This row of numbers contains the dimension of each basin which was estimated using:

(3) $$D_{i}=\exp\left(-\sum_{k=1}^{M}\eta_{k}\log\eta_{k}\right)\text{ where }\eta_{k}=\frac{\lambda_{k}}{\sum_{j=1}^{M}\lambda_{j}}$$

and {λ_*k*_} is the eigenvalue spectrum of the covariance matrix for the *i*th PAMM feature. As you can clearly see all the algorithms do a good job of projecting clusters that have an estimated dimensionality that is less than around seven. These features appear as a single cluster in the low dimensional space. It is those features that have an estimated dimension that is higher than around seven that represent a problem. The projections of points from these clusters are often spread across multiple separated clusters, which makes it difficult to realize that these configurations are all part of a single basin.

It is interesting to note from Figure 3 how strongly the projection generated using Laplacian Eigenmaps differs from the others. The projection generated using this algorithm has the light green motif separated strongly from all the other motifs, which appear squeezed together. This same squeezing together of some of the motifs and pulling apart of others is not observed in the other representations of the trajectory. The representative structure for the light green motif offers a tantalizing explanation as to why this particular behavior might be observed for this particular algorithm. The light green motif is the only structure containing no secondary structure content. One might therefore suppose that this motif corresponds to a random coil, that the diffusion distance, which Laplacian Eigenmaps endeavors to reproduce when it constructs a projection, between these states and the other folded configurations might well be quite large, and that the transitions between this random coil state and the other folded states might thus be quite infrequent. As we will show in what follows it is not clear that this interpretation is correct, however.

**Figure 4:**
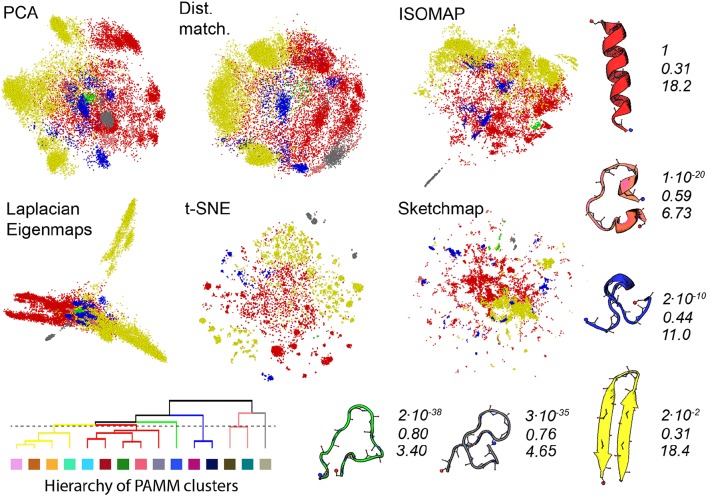
As discussed in the text, we can use an agglomerate procedure based on the error in the KDE procedure that is at the heart of PAMM to construct the hierarchy shown in the bottom left corner of this figure for the PAMM clusters that were identified and shown in Figure 3. This hierarchy would seem to indicate that we can reduce the 11 PAMM clusters shown in Figure 3 down to 6 macro clusters. In the projections above the points are therefore colored according to the particular macro cluster the PAMM algorithm identities each of the high dimensional configurations to lie within. As in Figure 3 representative and similarly-colored configurations from each of the various clusters are shown below and to the right of the projections together with scaled values for determinant of the covariance matrices for each of the clusters (**Top**), ratios of the sum of the largest two eigenvalues of these matrices to the sum of all the eigenvalues of these matrices (**Middle**) and the estimated dimensionality of each of the features (**Bottom**).

Gasparotto et al. (2018) discuss how bootstrapping can be used to judge the statistical significance of the clusters identified by PAMM. Furthermore, in analysing the errors in this way a distance between clusters that determines whether or not clusters get merged in some of the 41 bootstrap samples that we took from the trajectory can be defined. We have used this distance measure in Figure 4 to generate a tree-like plot that illustrates the results of a hierarchical clustering procedure performed on the eleven clusters that were identified in Figure 3. The clusters that are connected in this plot are those that are likely to be merged in the bootstrap samples. We thus also re-show the projections generated using each of the algorithms in Figure 4 but this time we have reduced the number of PAMM clusters from eleven to six by using the connectivity that is identified in the tree diagram.

The PAMM analysis shown in Figure 4 makes clear that the free energy landscape for this protein contains two broad funnels and three additional, much-smaller funnels. The beta-hairpin and alpha-helical configurations lie at the bases of the two broad funnels while the three narrower funnels have three unfolded structures at their base that have much higher energies. We can speak of the size of the funnels because we have, once again, performed an analysis of the covariance matrices and because the determinant of the clusters that contain the alpha-helix and beta-sheet are both considerably larger than those of the other identified features. The other aspects of this analysis demonstrate that if we take the ratio of the sum of the two largest eigenvalues of the covariance matrix to the sum of all the eigenvalues of this matrix we find that many of the fluctuations that take place within these two funnels take place along directions that are orthogonal to the direction of the eigenvectors that correspond to these two largest eigenvalues of the funnel. Furthermore, the estimated dimensionalities of these two features are substantial.

When we look at the various projections in Figure 4 we see that all the algorithms do a good job of separating the red configurations that come from the funnel that has the alpha helix at is base from the yellow configurations that come from the funnel that has the beta sheet at its base. Furthermore, the fact that these two features have a larger spatial extent than the other clusters identified by PAMM is clear from the way these features are projected using all the different algorithms. Having said all that though there is no way that one would be able to identify these two features in the landscape if one were just given the projections generated using one of these algorithms. In all these various representations of the trajectory, the red and yellow points appear divided up into multiple separate clusters. The most dramatic example of this is in the projection generated using Laplacian Eigenmaps which divides the yellow points between two very distinct clusters. It is clear from a comparison of the projections in Figures 3 and 4 that the configurations in these two clusters are separated in all the other projections of the trajectory. Furthermore, the hierarchy of clusters shown in Figure 4 also indicates that these two clusters are likely to be separate features. It may well be, therefore, that the rate of transition between these two parts of configuration space is slow because there is perhaps a kinetic trap on the folding funnel for the beta hairpin. This observation does, however, raise an interesting question when it comes to selecting which dimensionality reduction algorithm to use when constructing a projection. Using the slow degrees of freedom to construct a low-dimensional representation of the data makes physical sense but it may well be that the projections generated using algorithms that work by constructing a low dimensional representation of a trajectory in which the dissimilarities between trajectory frames are reproduced may give one a clearer sense of the various different structural possibilities in the ensemble.

**Figure 5:**
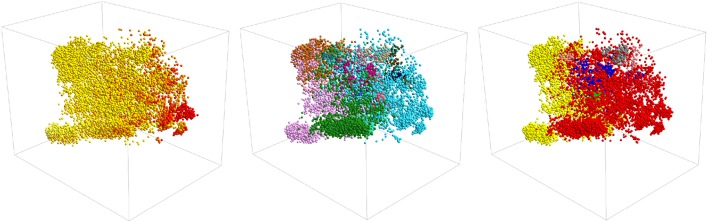
A three-dimensional projection of the trajectory that was generated using PCA. The positions of projections in the three panels above are all identical. In the left-most panel, however, points are colored in accordance with their secondary structure as they are in Figure 1. In the middle panel, they are colored as they are in Figure 3 and in the right panel they are colored as in Figure 4.

It is interesting to ask if we can construct a clearer visualization of the structure in the data by producing a three-dimensional projection. Figure 5 shows three representations of a three dimensional PCA projection of the trajectory with the points colored as in Figures 1, 3, 4. It is clear from this figure that the points in this three dimensional PCA projection are spread out over all three coordinates and certainly not split into distinct clusters. Furthermore, when it comes to distinguishing configurations with different secondary structures the projection is OK but there is still a substantial overlap between the regions of space where the structure has a lot of alpha-helical content and the regions of space where the structure more closely resembles a beta-hairpin as was the case for the two dimensional projection in Figure 1. In addition, each of the various PAMM features identified in Figures 3, 4 does not appear as a single cluster that is well separated from each of the other features. Instead, the points belonging to each of these features appear split between multiple apparently distinct clusters much like they appeared in the two-dimensional projections shown in Figures 3, 4. In short, a three-dimensional projection of this trajectory does not provide much greater insight than the two-dimensional projections that we have shown thus far and is considerably harder to visualize and interpret.

## 5. Conclusions

In the preceding sections, we have analyzed a molecular dynamics trajectory for a short protein molecule using a number of different dimensionality reduction algorithms. The results we have are in some senses reassuring as all the algorithms do a reasonable job when it comes to giving a representation of the trajectory that gives a sense of the structural diversity that one observes in the trajectory. In all the projections if two configurations have markedly different structures they are projected in different parts of the low dimensional space. Furthermore, configurations that are structurally similar are for the most part projected close together. In other words, even projections constructed using the easier to apply dimensionality reduction algorithms such as PCA and MDS, which have no parameters that need to be tuned, can provide one with a useful visualization of the high-dimensional data.

When one of these dimensionality reduction algorithms clearly outperforms the others it is often because the data has some structure that only one of the algorithms can recognize. For instance, isomap will outperform PCA when it comes to projecting data that lies on a curved manifold because PCA assumes the data lies on a linear manifold in the high dimensional space. The fact that all the algorithms perform similarly well and that no algorithm outshines the other thus perhaps simply reflects the fact that we do not fully understand how the trajectory data is distributed across the high-dimensional space. In other words, none of the data distribution models underlying these various algorithms provides a complete description of the structure of the data in the high-dimensional space. It seems that the data does not all lie on a low-dimensional linear or non-linear manifold and similarly there perhaps isn't a single length scale that separates configurations that lie in different basins in the free energy landscape. Perhaps then, given that all these algorithms are imperfect, the appropriate strategy for analysing an MD trajectory is to try something similar to the approach that has been taken in this paper. In short, analyze the trajectory using a range of different dimensionality reduction and clustering algorithms and consider what the result from each analysis is telling you by comparing the results obtained.

## Data Availability

The trajectories for the immunoglobulin binding domain B1 of protein G that we have analyzed within this article can be found online at https://github.com/cosmo-epfl/sketchmap/tree/master/examples/protein.

## Author Contributions

GT designed the work and wrote the text of the paper. PG analyzed the trajectories and produced the figures.

### Conflict of Interest Statement

The authors declare that the research was conducted in the absence of any commercial or financial relationships that could be construed as a potential conflict of interest.
