# Supplementary material for: Using Dimensionality Reduction to Analyze Protein Trajectories
Source: Front Mol Biosci. 2019 Jun 19;6:46. doi: 10.3389/fmolb.2019.00046 (PMC6593086; doi:10.3389/fmolb.2019.00046)
Supplement: Supplementary file 1 [file Data_Sheet_1.PDF]

Supplementary Material

In this document we provide some details about how the analysis and the figure in the main text were produced. We used Wolfram Mathematica 11.3 to produce the scatter plots while Adobe Illustrator CC 2019 was used to assemble all the final images.

1 FIGURE 1

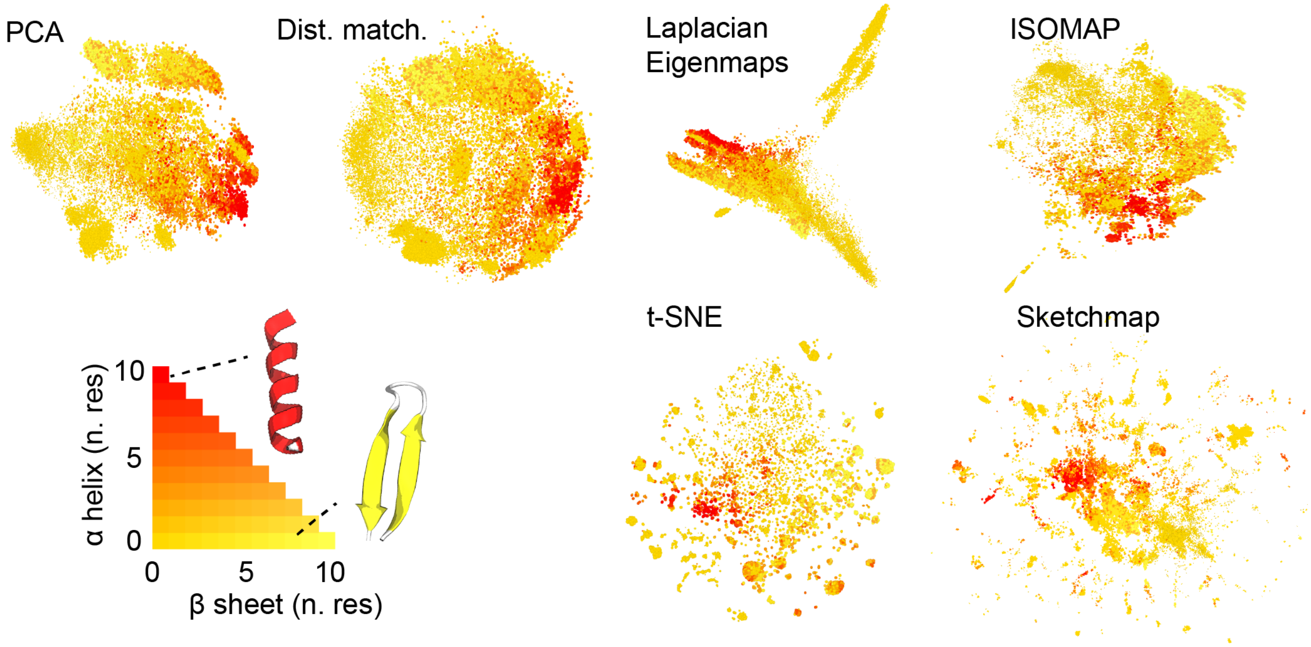

Figure S1. Projections of the trajectory using each of the various dimensionality reduction algorithms with points colored in accordance with the secondary structure.

1.1 Measuring the secondary structure content

Starting from the PDB trajectory, we used DSSP (*http://swift.cmbi.ru.nl/gv/dssp/HTML/distrib.html*) for the secondary structure assignment of each residue. For reference, the DSSP secondary structure classification is as follows: B, isolated β-bridge; E, extended strand; G, 3_{10}-helix; H, α-helix; I, π-helix; T, turn; S, bend; C, loop, irregular element, or none of the above ("coil"). The following bash script was used to prepare the inputs and to process the output from the DSSP analysis:

```
#!/bin/bash

for i in {1..25311}; do
    # save each frame in a temporary file
    head -$(echo "$i * 257"| bc) traj.pdb  | tail -257 > temp.pdb
    # run DSSP and output only the secondary structure assignment
    mkdssp -i temp.pdb | tail -16 | awk '{print substr($0,14,2)}' \
            >> ss.tmp
    # count the helical content
```

```
    alphanum=$(grep -c '[G,H]' ss.tmp)
    # count the strand content
    betanum=$(grep -c E ss.tmp)
    # store to a file
    echo $alphanum $betanum >> ss.dssp
    rm temp.pdb ss.tmp
done
```

Listing 1. Secondary structure assignment from a PDB trajectory using DSSP and bash.

This script generates a file called *ss.dssp* that contained the number of residues with an alpha helical shape in the first column and the number of residues with a beta sheet structure in the second column. This file was used when creating the colours of the points in all the projections.

1.2 PCA projection

The PCA projection was generated by using the following calls to the *sklearn.decomposition.PCA* library and Python 2.7. The script below shows how the image shown in the top left of Fig. 1 was generated from the trajectory. Note that, as suggested in the literature Yuguang et al. (2005); Konrad (2006); Altis et al. (2007), instead of using the dihedral angles directly in the PCA, we used their projection on the unit circle.

```
from numpy import *
from sklearn.decomposition import PCA

X=loadtxt(phi_psi.dat)
pca=PCA(n_components=2)
pca.fit(X)
J=pca.transform(X)
savetxt("PCA.proj", J)
```

Listing 2. PCA projection using scikit-learn.

1.3 Distance Matching

The Distance Matching projection was generated using the program *dimred*, which is part of the Sketch-map suite that is downloadable from *https://github.com/cosmo-epfl/sketchmap*. Starting from the list of the backbone ϕ, ψ dihedral angles we obtained the projection with the following command:

```
dimred -D 30 -d 2 -center -pi 6.283185307179586476925286766 56 \
       -preopt 100 < phi_psi.dat > DM.proj
```

Listing 3. Distance matching using *dimred*.

Multi-dimensional scaling is performed to get the starting intial positions followed by an iterative optimization of 100 stpes of conjugate gradient (*-preopt 100*).

1.4 Lapclacian Eigenmaps

Spectral Embedding is an approach for calculating a non-linear embedding. Scikit-learn implements Laplacian Eigenmaps, which finds a low dimensional representation of the data using a spectral decomposition of the graph Laplacian. The Laplacian Eigenmaps projection was generated using the *sklearn.manifold.spectral_embedding* library and Python 2.7. The script below shows how the projection in Fig. 1 was produced from the trajectory.

```
from numpy import *
from sklearn import manifold

X=loadtxt(distancematrix.dat)
n_neighbors=15
n_components=2
le = manifold.SpectralEmbedding(n_components=n_components, \
                                n_neighbors=n_neighbors, \
                                affinity='precomputed')
J=le.fit_transform(X)
savetxt("LE.proj", J)
```

Listing 4. Lapclacian Eigenmaps using scikit-learn.

Notice that in order to account for the periodicity of the dihedral angles a precomputed distance matrix is given as input to the library.

1.5 ISOMAP projection

The ISOMAP projection in Fig. 1 was generated using the following calls to the *sklearn.manifold.Isomap* library and Python 2.7. Here is the script that was used to obtain the projection from the trajectory:

```
from numpy import *
from sklearn import manifold

X=loadtxt(phipsi.dat)
n_neighbors=15
n_components=2

isomap = manifold.Isomap(n_neighbors, n_components=2)

J=isomap.fit_transform(X)
savetxt("ISOMAP.proj", J)
```

Listing 5. ISOMAP projection using scikit-learn.

We used the default settings when defining the geodesic distance and the method for finding shortest path between two points.

1.6 t-SNE projection

To produce the t-SNE projection we use the library *sklearn.manifold.TSNE* and Python 2.7. The script used to obtain the projection shown in Fig. 1 is as follows:

```
from numpy import *
from sklearn import manifold

X=load(distancematrix.dat)
n_neighbors=15
n_components=2

tsne=manifold.TSNE(n_components=2, perplexity=50.0, \
                   early_exaggeration=10.0, \
                   learning_rate=100.0, n_iter=3500, \
                   n_iter_without_progress=300, \
                   min_grad_norm=1e-07, metric="precomputed",\
                   init='random', method="barnes_hut", angle=0.5)
J=tsne.fit_transform(X)
savetxt("TSNE.proj", J)
```

Listing 6. t-SNE projection using scikit-learn.

As was the case for the Lapclacian Eigenmaps we provided a pre-computed distance matrix to account for the periodicity of the dihedral angles.

1.7 Sketch-map projection

The sketch-map projection was generated using the libraries part of the sketch-map suite downloadable from *https://github.com/cosmo-epfl/sketchmap*. The procedure used to generate the projection is already described in details in Ref. Ardevol et al. (2015). In essence, however, 1000 landmarks points were selected from the initial trajectory using FPS, which is implemented in the program *dimlandmark*. An optimal 2D sketch-map projection for these landmarks was then produced using the program *dimred*. Once a projection for each of the landmarks was found, the remainder of the trajectory was projected into the sketch-map space using *dimproj*. The commands used to produce the sketch-map projection shown in Fig. 1 is as follows:

```
# select 1000 landmarks using FPS
dimlandmark -D 30 -n 1000 -pi 6.283185307 \
          -mode minmax -w -lowmem < phipsi.dat \
          > lands1000.HD

# running a preliminary iterative metric MDS
grep -v \# lands1000.HD | \
     dimred -vv -D 30 -d 30 \
          -pi 6.283185307 \
          -center -preopt 100 > lands1000.imds 2>>log
grep -v "#" lands1000.imds | awk '{print $1, $2}' > tmp
grep -v \# lands1000.HD | \
     dimred -vv -D 30 -d 30 \
          -pi 6.283185307 \
```

```
            -center -preopt 100 -fun-hd 6,8,8 \
            -fun-ld 6,2,8 -init tmp > lands1000.ismap 2>> log

GW=$(awk 'BEGIN{maxr=0} !/#/{r=sqrt($1^2+$2^2); \
         if (r>maxr) maxr=r} END{print maxr*1.2}' lands1000.imds)
NERR=$(awk '/Error/{print $(NF)}' lands1000.imds | tail -n 1)
SMERR=$(awk '/Error/{print $(NF)}' lands1000.ismap | tail -n 1)

# running sketch-map
IMIX=1.0
MAXITER=10
for ((ITER=1; ITER<=$MAXITER; ITER++)); do
   MDERR=$NERR
   if [ ! -e $FILELD.gmds_$ITER ]; then
      grep -v \# lands1000.HD | \
        dimred -vv -D 30 -d 30 \
            -pi 6.283185307 \
            -center -preopt 50 -grid $GW,21,201 \
            -fun-hd 6,8,8 -fun-ld 6,2,8 -init tmp -gopt 3 \
            -imix $IMIX > lands1000.gmds_$ITER 2>>log
   fi
   grep -v "#" lands1000.gmds_$ITER | awk '{print $1, $2}' > tmp
   GW=$(awk 'BEGIN{maxr=0} !/#/{r=sqrt($1*$1+$2*$2); \
        if (r>maxr) maxr=r} END{print maxr*1.2}' lands1000.gmds_$ITER)
   # get the residual error
   NERR=$(awk '/Error/{print $(NF)}' lands1000.gmds_$ITER | \
          tail -n 1)
   IMIX=$(echo "$IMIX $SMERR $NERR" | \
          awk '{new=$2/($2+$3); if (new<0.1) new=0.1; \
          if (new>0.5) new=0.5; print new*$1 }')
   if [ ` echo $MDERR $NERR | \
        awk -v i=$ITER '{ if (i>1 && (($1-$2)/$2)*(($1-$2)/$2)<1e-4)\
        print "done"; else print "nope";}' ` = "done" \
      ]; then ((ITER++)); break;
   fi;
done

# Doing final fit
((ITER--))
grep -v "#" lands1000.gmds_$ITER | awk '{print $1, $2}' > tmp
grep -v \# lands1000.HD | dimred -vv -D 30 -d 30 \
            -pi 6.283185307 \
            -center -preopt 100 \
            -grid $GW,21,201 -fun-hd 6,8,8 \
            -fun-ld 6,2,8 -init tmp -gopt 10 > lands1000.gmds 2>>log

# Output only the final projection
grep -v "#" lands1000.gmds | awk '{print $1, $2}' > lands1000.skmap

# Out-of-sample embedding
dimproj -D 30 -d 2 -P lands1000.HD -p lands1000.skmap \
        -pi 6.283185307 -w -grid $GW,21,201 -fun-hd 6,8,8 \
        -fun-ld 6,2,8 -cgmin 3 < phipsi.dat > SKMAP.proj
```

Listing 7. Sketch-map workflow.

This script generated a file called *SKMAP.proj* which contained the low dimensional projection of the high-dimensional backbone dihedrals feature space.

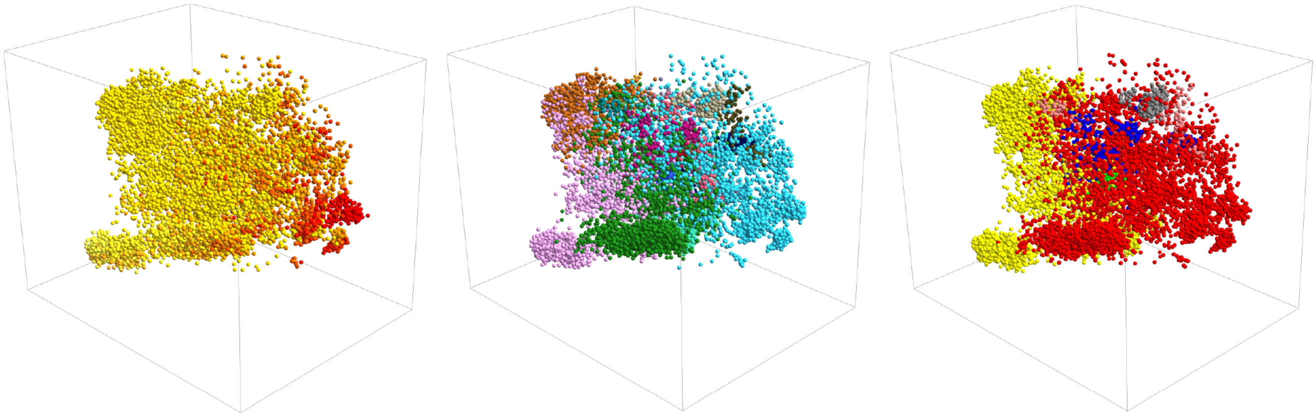

Figure S2. PCA projection in 3D. Points are colored in accordance with the secondary structure, the PAMM clusters and the macro-clusters described in the main text.

2 3D PCA

The 3D PCA projection was generated using the *sklearn.decomposition.PCA* library and Python 2.7, as described previously for the 2D case. We used to following script to generate the 3D embedding:

```
from numpy import *
from sklearn.decomposition import PCA

X=loadtxt(phi_psi.dat)
pca=PCA(n_components=3)
pca.fit(X)
J=pca.transform(X)
savetxt("PCA.proj", J)
```

Listing 8. 3D PCA projection using scikit-learn.

3 PAMM CLUSTERING

PAMM clustering was performed directly in the high-dimensional backbone dihedrals feature space using the FORTRAN90 code available at *https://github.com/cosmo-epfl/pamm*. which was described in Gasparotto et al. (2018) using the. The clustering procedure has been applied to the full original trajectory from Ref. Ardevol et al. (2015) using the following command:

```
pamm -d 30 -fpoints 0.1 -qs 0.95 -bootstrap 41 < fulltraj.phipsi
```

Listing 9. PAMM HD clustering.

The PAMM code allows you to use periodic variables. In this case the periodicity of these variables have been hard coded into the particular version of the software used. When using other versions of the code, however, you may have to specify that the input variables are periodic by using a suitable command line flag.

PAMM produced a series of file: *out.pamm* (a Gaussian Mixture Model describing the Probability Density Function generating the data), *out.grid* (a sub-grid sampled using FPS approximating the whole dataset), *out.voronoislinks* (the Voronoi assignment that allows one to reconstruct the original dataset from the FPS grid), *out.bs* the overlap between clusters estimated from the bootstrapping. More details on PAMM can be found in Ref. Gasparotto et al. (2018).

REFERENCES

Altis, A., Nguyen, P. H., Hegger, R., and Stock, G. (2007). Dihedral angle principal component analysis of molecular dynamics simulations. *The Journal of Chemical Physics* 126, 244111. doi:10.1063/1.2746330

Ardevol, A., Tribello, G. A., Ceriotti, M., and Parrinello, M. (2015). Probing the unfolded configurations of a β-hairpin using sketch-map. *Journal of Chemical Theory and Computation* 11, 1086–1093. doi:10.1021/ct500950z. PMID: 26579758

Gasparotto, P., Meißner, R. H., and Ceriotti, M. (2018). Recognizing local and global structural motifs at the atomic scale. *Journal of Chemical Theory and Computation* 14, 486–498. doi:10.1021/acs.jctc.7b00993. PMID: 29298385

Konrad, H. (2006). Comment on: "energy landscape of a small peptide revealed by dihedral angle principal component analysis". *Proteins: Structure, Function, and Bioinformatics* 64, 795–797. doi:10.1002/prot.20900

Yuguang, M., H., N. P., and Gerhard, S. (2005). Energy landscape of a small peptide revealed by dihedral angle principal component analysis. *Proteins: Structure, Function, and Bioinformatics* 58, 45–52. doi:10.1002/prot.20310
